# User Perspectives on Conversational Agents for Preventive Alcohol Self-Help: Qualitative Exploratory Study

**DOI:** 10.2196/86752

**Published:** 2026-07-14

**Authors:** Marissa De Vries, Melissa Begeman, Marloes Derksen, Anna Goudriaan, Matthijs Blankers

**Affiliations:** 1 Department of Research, Arkin Mental Health Care, Amsterdam Institute for Addiction Research, Klaprozenweg 111, Amsterdam, 1033NN, The Netherlands, +316 187 82 819; 2 Department of Medical Informatics, eHealth Living & Learning Lab Amsterdam, University of Amsterdam, Amsterdam University Medical Centers, Amsterdam, The Netherlands; 3 Department of Psychiatry, Amsterdam University Medical Centers, Amsterdam, The Netherlands; 4 Department of Drugs, Institute of Mental Health and Addiction, Trimbos Institute, Utrecht, The Netherlands; 5 Tranzo Scientific Center for Care and Wellbeing, Tilburg School of Social and Behavioral Sciences, Tilburg University, Tilburg, The Netherlands

**Keywords:** conversational agents, preventive health care, self-help, alcohol use, qualitative study

## Abstract

**Background:**

Alcohol use remains a major public health concern, and although preventive alcohol self-help interventions aim to support individuals in need of indicated prevention, they continue to face challenges related to low engagement and high attrition. Chatbots, also known as conversational agents (CAs), powered by artificial intelligence, may enhance engagement by offering personalized guidance and 24/7 availability. Yet, user perspectives on CAs in preventive alcohol self-help care remain largely unexplored.

**Objective:**

This paper aimed to gain insight into the diverse factors shaping the adoption of CAs in preventive alcohol self-help by qualitatively exploring users’ expectations and concerns, complemented by descriptive questionnaire data on perceived usefulness, intention to use, comfort, and trust.

**Methods:**

This qualitative, exploratory study with a small purposive sample (N=12) included adults recruited from a Dutch digital alcohol self-help intervention and a client advisory board. Data were collected during an online focus group (n=8, 66.7%), 4 semistructured interviews (n=4, 33.3%), and a brief questionnaire administered to all participants in March 2025 and April 2025. The qualitative data were analyzed inductively using thematic analysis.

**Results:**

Qualitative findings showed that participants appreciated the 24/7 availability, practical guidance, and nonjudgmental stance of CAs but doubted their capacity for genuine empathy and authenticity. Participants emphasized that the conversational tone was important: CAs should be supportive without being overly agreeable and should be confrontational once trust is established. Concerns about undue influence arose occasionally, while privacy was viewed by 1 (8.3%) participant as a major issue. Descriptive questionnaire results showed that 11 (92%) participants had prior experience with CAs, whereas only 6 (50%) had experience with digital health interventions. Most participants considered CAs potentially useful for reducing alcohol use, yet few expressed strong intentions to use one themselves. Comfort and trust ratings were largely neutral, reflecting a cautious but generally open attitude toward CA use.

**Conclusions:**

CAs appear to hold promise as complementary tools for preventive alcohol self-help, although they are not perceived as replacements for human contact. Future design efforts should emphasize personalized interactions; an appropriate conversational tone, supportive yet not overly agreeable; and transparent communication about data use to enhance engagement and acceptance. The findings are descriptive and are not intended to be broadly generalizable.

## Introduction

Although global alcohol consumption has declined since 2010, alcohol use remains a significant public health concern [1]. Alcohol use is associated with increased risks of physical illness, psychological distress, and adverse social outcomes [2]. Despite these consequences, globally only around 1 in 6 individuals with alcohol use disorder receives any form of treatment [3]. This treatment gap is driven by a range of factors, including limited availability of services, inadequate screening and referral procedures, and stigma surrounding alcohol-related problems [4].

In response to the limited availability of services, digital interventions have emerged as promising tools to increase access to care. These interventions, delivered via websites or mobile apps, can be used independently or alongside professional treatment. They are typically low-threshold, flexible in timing and location, and have shown small to moderate effects in reducing alcohol use and improving mental health outcomes [5,6]. There is growing recognition that digital interventions should not only target individuals with alcohol use disorders but also those engaging in risky drinking behavior. Preventive digital programs can reach this broader group early, potentially reducing harm before dependence develops [7,8].

Preventive digital interventions can vary in intensity, and a key distinction concerns the degree of guidance. Guided interventions—those that include contact with a therapist or coach—tend to show higher adherence than fully automated self-help formats [5,9,10]. This suggests that some form of personalized feedback or interaction is important for maintaining engagement. Conversational agents (CAs), also known as chatbots, offer a potential solution to deliver such guidance in a scalable way. A CA is an artificial intelligence (AI) system designed to engage users in natural, humanlike dialogue. Preliminary evidence suggests that CAs can enhance user satisfaction and engagement compared to unguided digital interventions and may even support reductions in depressive symptoms [11-13].

Although evidence for the effectiveness of CAs in digital mental health care is increasing, it is also important to understand users’ perspectives on these technologies. A recent qualitative study with users of a CA for anxiety reported that participants valued its accessibility but expressed concerns about the agent’s ability to demonstrate empathy and maintain privacy. Many participants also emphasized the need for human supervision [11]. A review highlighted that factors such as personalization, reliable responses, and ease of use are important for user engagement with these technologies [14]. Additionally, although some users remain skeptical about the role of CAs in providing health advice, they appear somewhat more open to using them as a supplement to mental health support [15]. Reviews further emphasize that personalization, reliability, and transparent communication are central to sustained engagement [14].

Although existing research provides important insights into the general use of CAs for mental health, much less is known about their potential role in alcohol-related prevention and self-help. Alcohol consumption involves distinct behavioral, social, and motivational dimensions that may influence how users perceive and interact with automated support. Understanding these perceptions is important for assessing whether CAs can effectively complement existing preventive self-help approaches and for identifying factors that may facilitate or hinder their adoption. This study therefore examines the experiences, expectations, and concerns of participants in a Dutch preventive alcohol self-help program and of members of a client advisory board, focusing on their perspectives on CAs in digital alcohol prevention and the reasons why they would or would not use such tools. Given the limited existing evidence, the study adopted an exploratory qualitative design without predefined hypotheses or preregistration, with the goal of generating inductive insights to inform the future development and evaluation of CAs in digital alcohol prevention interventions.

## Methods

### Study Design

This study used an exploratory qualitative design based on an online focus group, 4 semistructured interviews, and a brief questionnaire to gather contextual information. The focus group primarily generated interactive discussions around shared experiences and general expectations, while the individual interviews provided more nuanced and personal reflections, particularly regarding doubts about empathy, trust, and personal willingness to use CAs. The questionnaire was administered prior to the focus group and interviews to obtain participants’ independent views on CAs, uninfluenced by group dynamics, and to serve as a basis for more in-depth exploration during the discussions. The questionnaire data were used descriptively and for contextual triangulation of the qualitative findings, rather than for inferential analysis. Given the small sample size, the quantitative results (eg, mean scores) should be interpreted with caution and are intended to provide indicative insights only.

### Recruitment

The qualitative and quantitative components of the study included 12 participants recruited between March 2025 and April 2025 from a Dutch online self-help program for alcohol use and the client advisory board of a large mental health care institution (Arkin, the Netherlands). The self-help program consists of 6 evidence-based modules based on both motivational interviewing and cognitive behavioral therapy. Other intervention features include a support forum, a personal diary, and encouragement for users to log their daily substance use and craving in accordance with cognitive behavioral therapy principles.

Inclusion criteria were participation in Jellinek’s digital self-help alcohol intervention, familiarity with the program, proficiency in Dutch, and willingness to participate in an online group discussion or interview. Due to the anonymous nature of the self-help program, participants’ contact details were not initially available. However, individuals who expressed interest in participating in the study by responding to a forum message or clicking a banner within the self-help program were asked to provide their phone number or email address, enabling the researchers to provide more information and formally invite them to the focus group. A total of 24 participants provided contact details, all of whom were contacted multiple times to schedule participation. Of these, 2 (16.7%) participants did not speak Dutch, 6 (50%) were unable or unwilling to join an online group discussion or interview, and 7 (58.3%) did not respond to phone calls or emails. Ultimately, 8 (66.7%) participants from the self-help community joined the focus group. In addition to the focus group, 4 semistructured interviews were conducted. Three interviews were held with members of Arkin’s client advisory board, and 1 was conducted with another self-help user who did not want to join a group discussion but was willing to participate in an interview. Because of the limited number of participants (n=12, 100%), formal data saturation could not be established; however, recurring patterns and overlapping themes emerged across the focus group and interviews. This suggests a degree of thematic consistency, even if full saturation was not attained [16].

All participants completed a questionnaire prior to the focus group and interviews and received a €45 (€1=US $1.14 as of June 23, 2026) gift voucher as compensation.

### Data Collection Procedures

Data were collected between March 2025 and April 2025 through 1 online focus group session (n=8, 66.7%) and 4 online semistructured interviews (n=4, 33.3%). The focus group followed a semistructured discussion format exploring attitudes toward the integration of a CA. The focus group lasted approximately 3 hours, although it was planned for 2.5 hours. The same thematic areas were addressed in the individual interviews, allowing deeper reflection and more personal nuance. The interviews lasted approximately 1.5 hours. The focus group was moderated by an independent researcher, a female psychotherapist (MSc) with prior experience in moderating focus groups. She had no relationship with any participants. She was independent because she did not have assumptions or personal interests related to the study, which was important to minimize the risk of leading questions or undue influence. The primary researcher (first author) was present during the focus group in a nonparticipatory role. She introduced herself as the researcher of this project, explained why an independent person moderated the focus group, and explained the rationale for conducting this research. The researcher was also present to address potential procedural questions from participants and to become familiar with the data. This allowed the researcher to gain a contextual understanding of the discussions, including tone and group dynamics, which may not be fully captured in written transcripts. The researcher also made field notes during the focus group and interviews.

Prior to the focus group and interviews, participants were asked to complete a short, researcher-developed questionnaire about their prior experiences with CAs and their perspectives on how these could be used in the self-help program. All participants were asked for what purposes they would use a CA after being given several examples of possible functions. They were invited to write down as many desired functions as they wished. The questionnaire also included several demographic items (Table 1). The questionnaire was pretested by researchers involved in the study prior to data collection to ensure that it functioned properly and that the wording was comprehensible for individuals with varying levels of language proficiency. Several questionnaire items were also discussed during the focus group and interviews to gain deeper insight into participants’ responses and to stimulate group-level discussion (Checklist 1).

**Table 1.** Characteristics of participants (N=12).

Characteristics	Participants
Gender, n (%)	
Men	8 (66.7)
Women	4 (33.3)
Age (years), mean (SD)	61.1 (8.3)
Education level, n (%)	
Lower	3 (25.0)
Higher	9 (75.0)
Country of birth, n (%)	
The Netherlands	11 (91.7)
Belgium	1 (8.3)
Previous addiction treatment, n (%)	
Yes	6 (50.0)
No	6 (50.0)
Previous experience with digital interventions^a^, n (%)	
Yes	6 (50.0)
No	6 (50.0)

a Excludes the Jellinek digital alcohol self-help intervention.

### Data Analysis

The qualitative data were analyzed using inductive thematic analysis [17,18]. This method was selected for its suitability for generating in-depth, exploratory insights into participants’ experiences, perceptions, and attitudes regarding complex and potentially ambivalent topics such as AI in mental health care. In addition, the inductive approach enables researchers to maintain an open perspective toward the data. Alternative qualitative approaches, including grounded theory, content analysis, and framework analysis, were considered in consultation with a qualitative data supervision group before thematic analysis was selected as the most appropriate method, given the exploratory aim of the study.

The focus group and interviews were audio-recorded and transcribed verbatim. Transcripts were anonymized prior to analysis and were not returned to participants. The focus group and interviews were conducted in Dutch, and the quotes were translated into English and checked by a native speaker for accuracy.

In the first phase, the primary researcher (first author) familiarized herself with the data by repeatedly reading the transcripts of the focus group and interviews. An initial phase of open, inductive coding was then conducted, in which segments of the data relevant to the research question were identified. Given the nature of focus group and interview data, nonrelevant conversational content was not coded. Codes were generated at a semantic level, staying close to participants’ own words and meanings without imposing predefined categories.

After completion of the initial coding, codes were systematically compared and grouped based on similarities and patterns, resulting in the development of candidate themes. These themes were iteratively reviewed and refined in relation to both the coded extracts and the full dataset. The coding process and theme development were discussed with a second independent researcher (M Begeman). Discrepancies in interpretation were resolved through discussion until consensus was reached. A documentation process was maintained by documenting coding decisions, code definitions, and changes to the coding structure, supported by analytic memos and versioning within MAXQDA 2022 (VERBI Software GmbH). Participants did not provide feedback on the findings.

The questionnaire data were analyzed descriptively using means and SDs to provide contextual information about participants’ familiarity with and attitudes toward CAs.

### Ethical Considerations

All participants provided informed consent and received €45 as compensation for participating. The Medical Research Ethics Committees United determined that the study was not subject to the Dutch Medical Research Involving Human Subjects Act (Dutch: *wet medisch-wetenschappelijk onderzoek met mensen*) and granted approval (2024.1147). To minimize potential bias, the focus group was moderated by an independent facilitator who was not involved in the research project. The primary researcher has a background in clinical psychology and addiction research, with an interest in digital mental health. She was not involved in the development of the self-help program under study but is involved in planned future adaptations and improvements to the program. To minimize potential bias, the researcher adopted a nondirective role during the focus group, avoiding leading questions and allowing participants to freely express their perspectives.

## Results

### Participant Demographics

Participant characteristics are presented in Table 1. The sample consisted of 8 (66.6%) men and 4 (33.3%) women, with a mean age of 61.1 (SD 8.3) years. Previous research among users of the same self-help program has shown that the average age in this population is relatively high, at around 51 years [19]. Most participants had completed higher education, and half reported prior experience with digital interventions (excluding the Jellinek digital alcohol self-help intervention).

### Qualitative Data

The thematic analysis of the focus group data identified 2 overarching themes related to the use of CAs: motivating factors that encouraged participants to consider CAs as supportive tools and hindering factors that limited their willingness to engage with such systems. Within each theme, several subthemes captured both functional and experiential dimensions of how participants envisioned interacting with CAs in the context of a self-help program to reduce or quit their alcohol use.

Many of the themes raised by self-help participants during the focus group were echoed in the interviews with members of the advisory board, indicating a high degree of consistency across the 2 participant groups.

### Motivating Factors for CA Use

#### Immediate Support in Moments of Need

A central theme was the perceived value of CAs as a source of immediate support, particularly during moments of craving or relapse. Participants described situations in which traditional support options, such as friends, family, or health professionals, would be unavailable, noting that cravings often occur at night or during other moments of isolation. The promise of a tool that is available “anytime, anywhere” was repeatedly emphasized as a unique strength of CAs.

One participant explained the following:

When you wake up at 2:30 a.m. craving a drink, it would be great to have something right then.

For several participants, this possibility filled a gap that existing services or peer support could not reliably address. Others stressed that even if the CA could not “solve” the craving, the act of engaging in dialogue might provide enough distraction or motivation to resist the urge.

#### Information Source

Participants also emphasized the value of CAs as a reliable and comprehensive source of information. Many described using tools such as ChatGPT (OpenAI) in their daily lives as a quick reference point, highlighting their utility for clarifying questions about health, lifestyle, or substance use.

One participant shared the following:

I use ChatGPT like Google now.

Several saw this practical, informational function as more appealing than emotional support, which they preferred to seek from human contacts. The CA’s broad knowledge base was described as particularly useful for offering “unexpected angles” or “new ideas” that might not occur to the user.

One participant noted the following:

Sometimes it gives you ideas that make you think, wow. A little spooky, but interesting.

Such input was considered helpful for self-reflection, even when participants did not act directly on the advice.

#### Positive Design and Interaction Characteristics

Beyond specific functions, participants valued certain interactional characteristics of CAs. Among these were reliability (ie, being always available and never tired) and a nonjudgmental tone. Several participants contrasted this with human helpers, who might express disappointment, irritation, or fatigue in response to relapse disclosures. By contrast, the CA was viewed as a safer and more consistent space for sharing sensitive information.

One participant stated the following:

It doesn’t get angry with you or sigh when you’ve relapsed again.

In terms of design, participants expressed divergent preferences.

Some preferred a minimalist, text-only interface, emphasizing simplicity and focus:

Give me just text. No human-like figure.

Others felt that adding visual or customizable elements could make interactions more engaging, even playful:

I’d find it funny if you could create the character yourself. With a weird hat and glasses, so it feels like someone you know.

This design flexibility was highlighted as a motivating factor because it could adapt to different user needs and preferences.

### Hindering Factors for CA Use

#### Reluctance and Unfamiliarity

For several participants, reluctance to engage with CAs stemmed less from negative experiences and more from a lack of familiarity with the technology. The novelty of AI-based systems created hesitation, described by 1 participant as “cold feet.” This sense of uncertainty was tied to limited exposure and the perception that AI was still a “black box” or unknown territory. Others indicated that although they were curious, they preferred to observe developments from a distance before adopting such tools themselves. This hesitation therefore reflected not outright rejection but rather a cautious, wait-and-see attitude.

#### Privacy and Security Concerns

Privacy did not emerge as a dominant barrier in this study. Most participants distinguished between highly sensitive information, such as financial or medical records, which they would never entrust to a CA, and less sensitive disclosures about drinking behavior, which they perceived as relatively harmless.

One participant explained the following:

Who cares whether I drink or not? If that information leaks, that doesn’t bother me.

However, this general downplaying of privacy did not mean that all concerns were absent.

One participant raised questions about where their data might end up, mentioning companies or countries with geopolitical associations:

Which cloud does it end up in? To Elon Musk? The Chinese? The Russians?

These statements suggest that although privacy was not top of mind for everyone, uncertainties about data storage and control could still undermine trust if left unaddressed.

#### Lack of Human Empathy and Authenticity

One of the most frequently cited barriers was the perceived inability of CAs to replicate essential human qualities, particularly empathy. Several participants emphasized that emotional support during moments of vulnerability required a genuine human connection.

One participant stated the following:

When it comes to moral support, my experience is that it only works with people.

The absence of warmth or emotional resonance limited the value participants attributed to CAs in deeply personal situations, such as feelings of shame. Moreover, some participants found the communication style of existing chatbots unconvincing. Responses were described as “too smooth” or overly scripted, lacking the imperfections and nuances of human dialogue.

Rather than offering comfort, this polished style sometimes reduced credibility:

I don’t want a super smooth answer. I want something I can actually use.

These reflections highlight a tension around tone: responses that are overly smooth or pleasing may come across as inauthentic, making the CA feel less trustworthy or connected to the user’s real struggles.

#### Off-Putting Design and Potential Influence

Design elements were another source of hesitation. Although some participants appreciated the possibility of customizing avatars or visual interfaces, others described these features as deceptive.

The idea of interacting with a humanlike avatar provoked discomfort, with 1 participant remarking the following:

I’d feel betrayed if a fake person was sitting across from me.

Concerns about undue influence underscored the importance of transparency regarding the capabilities and limitations of CAs. Some worried that users with limited digital literacy or critical thinking skills might overrely on the system or accept its advice uncritically. The risk of undue influence underscored the importance of transparency about the limits of what CAs can and cannot do.

Finally, trust was repeatedly described as something that could not be assumed but had to be earned. Participants suggested that credibility would need to be built gradually, through consistent and reliable interactions over time. Without this foundation, early negative experiences could quickly erode the willingness to use the CA.

### Survey: Familiarity With and Opinions on CAs

**Figure 1.**
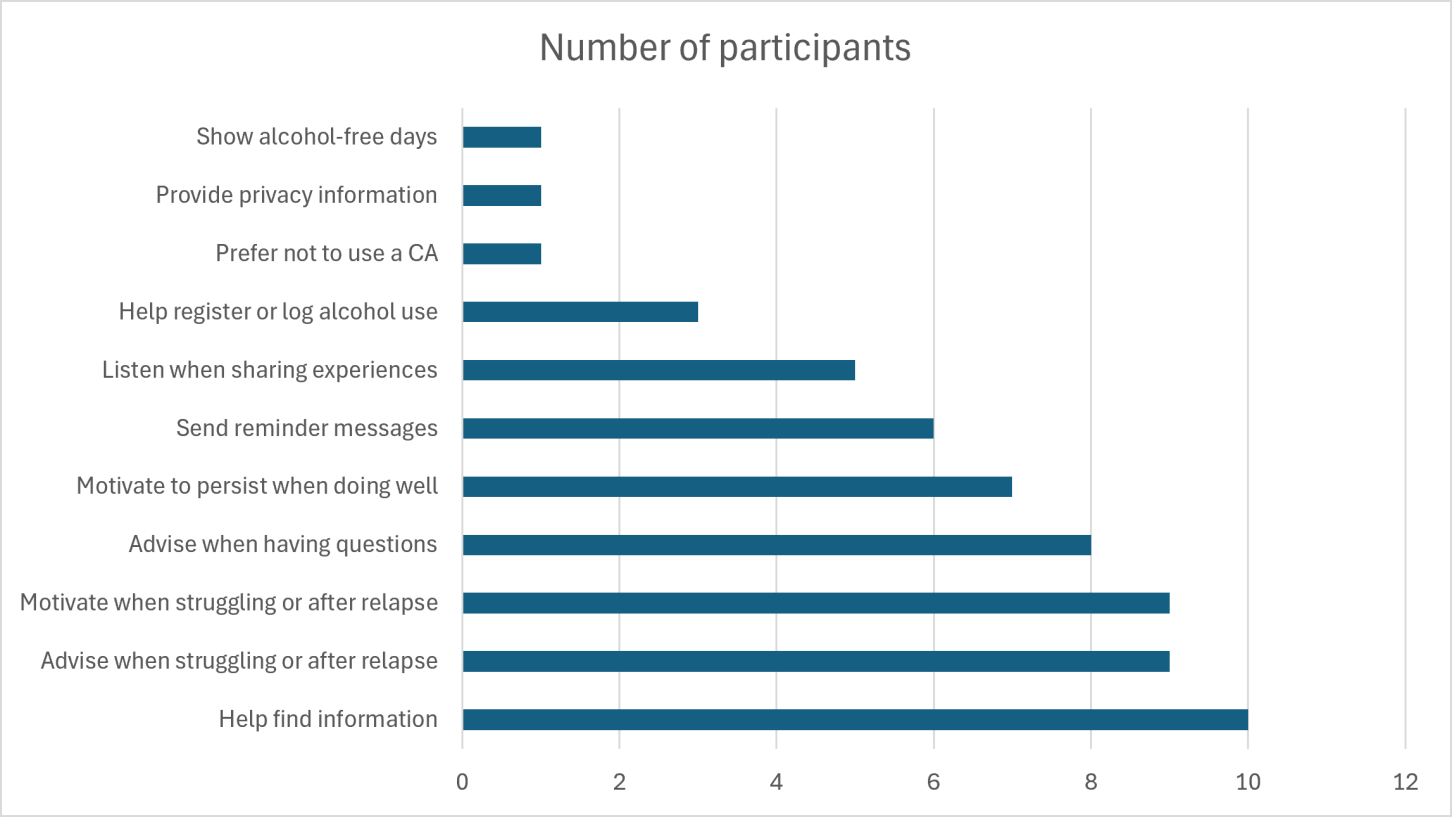
Desired functions of a conversational agent (CA) for alcohol self-help among participants (N=12).

Participants’ knowledge of and experiences with AI and CAs are presented in Table 2. The desired functions of a CA are presented in Figure 1.

The most frequently desired functions were helping users find information, providing advice, and offering motivation when struggling or after a relapse.

On a 5-point scale, participants reported moderate knowledge of AI (mean 3.0, SD 1.2) and perceived the usefulness of CAs for reducing alcohol use as fairly high (mean 3.8, SD 1.1). Comfort and trust levels were largely neutral (mean 3.0, SD 1.2 and mean 2.8, SD 0.9, respectively), while willingness to use a CA was mixed (mean 3.3, SD 1.3).

**Table 2.** Participants’ knowledge of and experiences with conversational agents (CAs; N=12).

Items	Response scale (5-point)	Participants, mean (SD)
Knowledge of AI^a^	“Very low” to “very high”	3.0 (1.2)
Usefulness of AI for reducing alcohol use	“Not useful” to “very useful”	3.8 (1.1)
Willingness to use a CA	“Definitely not” to “definitely yes”	3.3 (1.3)
Comfort discussing alcohol use with a CA	“Very uncomfortable” to “very comfortable”	3.0 (1.2)
Trust in a CA ability to give good advice	“Very low” to “very high”	2.8 (0.9)
Satisfaction with chatbots^b^	“Very dissatisfied” to “very satisfied”	3.6 (0.8)

a AI: artificial intelligence.

b Only participants with prior chatbot experience answered this question (n=11).

## Discussion

### Principal Findings

This qualitative study examined how users of a Dutch preventive alcohol self-help program and members of a client advisory board perceive the potential role of CAs in digital alcohol prevention. Across both qualitative and survey data, findings indicate a general perception of CAs as potentially useful, albeit accompanied by notable reservations regarding trust, authenticity, and willingness to adopt them.

Participants broadly valued the accessibility and functionality of CAs, particularly their around-the-clock support and practical guidance during moments of craving or relapse. They emphasized the appeal of an always-available, nonjudgmental companion that could offer both information and motivation without the social barriers often associated with human help. These qualitative perceptions were supported by survey results showing relatively positive evaluations of usefulness for reducing alcohol use (mean 3.8, SD 1.1), suggesting that participants recognize the potential functional value of CAs in preventive contexts.

Despite the perceived usefulness, both datasets consistently indicated ambivalence regarding personal uptake. Although survey responses indicated only moderate willingness to use a CA themselves (mean 3.3, SD 1.3), participants in the focus group and interviews expressed ambivalence about empathy and authenticity. This tension suggests that perceived usefulness does not directly translate into intended use.

Concerns about empathy and authenticity were central in both methods. In interviews and focus groups, participants questioned whether CAs could provide meaningful emotional understanding or whether responses might feel too “scripted” or “pleasing,” particularly in vulnerable situations. These concerns were also reflected in the survey data, with neutral trust ratings (mean 2.8, SD 0.9) and moderate comfort levels when discussing alcohol-related issues with a CA (mean 3.0, SD 1.2), indicating cautious attitudes toward relational aspects of CAs despite recognition of their functional benefits. Privacy concerns, in contrast, were mentioned less frequently and were generally not perceived as a major barrier.

Several factors that may facilitate engagement include trust, consistency, and flexibility in design. Participants viewed trust as something that must be earned gradually through reliable, transparent, and contextually appropriate interactions. The combination of practical functionality and emotional credibility appeared central to the willingness to use a CA in preventive care. Taken together, these findings provide a more nuanced picture of user perspectives on CAs in alcohol self-help: interest exists, but readiness to adopt CAs is conditional.

The study adds to existing knowledge by situating these insights specifically in a preventive context, where users are not seeking treatment but exploring support to maintain control over their drinking. This distinction helps explain the observed ambivalence, as CAs are perceived as promising complements rather than substitutes for human contact or established self-help methods.

### Comparison With Prior Work

The finding that accessibility, neutrality, and broad knowledge are seen as major advantages aligns with prior digital mental health research [20,21]. Our results extend this work by showing that these qualities may be particularly relevant in preventive contexts, where individuals often hesitate to seek formal treatment because of stigma or low perceived need. In such cases, the neutrality of a CA may not be a limitation but rather a functional strength, lowering the threshold for disclosure and support.

Personalization emerged as a strong desire in our sample, ranging from tailored interaction styles to flexible interface design. This resonates with broader evidence that adaptive systems can foster engagement and adherence [22]. At the same time, personalization brings to the forefront a central tension in digital health: the more interventions are tailored to individuals, the more data collection is required. Prior research has shown that increased data demands can create privacy concerns and reduce trust [15]. Interestingly, most of our participants downplayed privacy as an obstacle in hypothetical discussions. This aligns with previous research on the self-help intervention, which showed limited concern for ethical aspects of applying machine learning to adapt the digital intervention [23].

This discrepancy points to a possible gap between imagined use and real-world adoption. Our survey data support this interpretation. Although most respondents described AI as useful for reducing alcohol use (5=“very useful” and 3=“quite useful”), only a minority expressed strong intentions to use a CA themselves (3=“definitely yes”), with trust and comfort ratings largely neutral. These findings suggest that enthusiasm for CA functionalities in principle does not automatically translate into personal uptake.

Our findings also add nuance to debates about the emotional capacity of CAs. Previous studies have questioned whether they can provide empathy and called for human supervision [11]. Some participants in our study echoed these doubts, but others highlighted neutrality as preferable to human interaction, which can evoke feelings of disappointment or judgment. This suggests that the role of CAs may not be to replicate human warmth but to provide a complementary form of support in moments where neutrality is an advantage.

Finally, our results speak to the broader challenge of adherence in digital self-help programs. Dropout and inconsistent use remain persistent problems in unguided interventions [10]. The motivating factors identified by our participants—24×7 availability, nonjudgmental stance, and personalized feedback—mirror elements that prior research has associated with higher adherence in digital interventions, particularly studies showing that guidance and personalization increase completion rates [5,9,10,22].

### Broader Implications and Ethical Considerations

The findings highlight both practical and ethical implications for the design and implementation of CAs in preventive alcohol self-help. From a design perspective, participants expressed diverse preferences regarding visual appearance and interaction style. Some favored a simple, text-based interface, while others appreciated the idea of a more humanlike avatar or personalized appearance. However, across these differences, participants consistently emphasized that the *tone of* communication mattered most. The CA should respond in an empathetic, respectful, and nonjudgmental way, without becoming overly pleasing or superficial. Participants valued honesty and even confrontation when appropriate, but only once trust had been established. This suggests that CAs in this context should be capable of adjusting their interactional style over time, matching their tone and feedback to the user’s stage of engagement and level of trust.

Beyond design, and building on participants’ emphasis on 24×7 availability and low-threshold access, CAs may serve as complements to existing self-help or prevention programs by offering immediate support during moments when human help is unavailable. Integrating such systems into alcohol prevention and broader eHealth strategies could potentially increase accessibility, although this remains to be tested in practice. At the same time, the very features that make CAs attractive, such as their constant availability and responsiveness, may also introduce new ethical challenges. Participants’ concerns about undue influence and the lack of genuine empathy suggest that CAs are not merely neutral tools but may function as moral technologies that shape decision-making and redistribute responsibility within care relationships. For example, when users rely on a CA for advice during moments of vulnerability, questions arise regarding who holds responsibility for the outcomes of such interactions—whether this lies with the user, the developers, or the system itself.

### Limitations

Several limitations should be acknowledged. The sample was small, relatively homogeneous, and skewed toward older, higher-educated participants, which limits generalizability. However, previous research among users of the same self-help program has shown that the average age in this population is approximately 51 years [19], suggesting that the sample is broadly consistent with the program’s user base. Another limitation concerns recruitment. Because participants were informed that the study involved discussing AI-based tools, it is possible that the sample included individuals who were more interested in or open to technological innovations in health care than the average self-help user. This may have resulted in a group with relatively well-developed or positive opinions about CAs, potentially limiting the range of perspectives represented.

Finally, although recurring patterns and overlapping themes were observed across the focus group and interviews, formal data saturation could not be confirmed because of the limited number of participants. Future studies with a larger and more diverse sample could build on these findings to further explore differences in user expectations, trust development, and CA design preferences.

### Future Directions

Future research should replicate this study in larger and more diverse samples to verify whether these findings hold across different populations and drinking profiles and to achieve stronger data saturation. Observational and experimental studies involving actual interaction with CAs would allow researchers to examine how trust, comfort, and engagement evolve over time. Directly observing user-CA exchanges, for example, within existing preventive self-help platforms, could provide valuable behavioral data and reveal when and how users disengage.

It would also be valuable to explore how specific design features, such as text-only vs avatar-based interfaces and conversational tone, affect engagement. Finally, longitudinal research could clarify whether CAs can meaningfully support adherence and sustained behavior change over time, beyond initial curiosity or short-term use.

### Conclusions

This study shows that users see potential for CAs in preventive alcohol self-help, mainly because of their constant availability, practical guidance, and neutral stance. However, CAs are not perceived as replacements for human contact. Moreover, enthusiasm in principle does not automatically translate into willingness to use such tools, as doubts about empathy, authenticity, and trust remain. CAs should therefore be viewed as complementary support within broader self-help systems. Future design efforts should emphasize personalized design; an appropriate conversational tone, supportive yet not overly agreeable; and transparent communication about data use to enhance engagement and acceptance. Their future value will depend on design, user involvement, and real-world evaluation to strengthen trust and engagement.

## Supplementary material

10.2196/86752 Checklist 1 COREQ checklist.

## Abbreviations

AI: artificial intelligence
CA: conversational agent
